# Inhibition of the renin-angiotensin-aldosterone system prevents and cures atrial fibrillation: An overview of systematic reviews

**DOI:** 10.1097/MD.0000000000025559

**Published:** 2021-05-07

**Authors:** Zhixiang Yu, Dong Zhang, Qiuhe Ji, Fu Yi

**Affiliations:** a Department of Endocrinology and Metabolism; b Department of Cardiology, Xijing Hospital, The Air Force Military Medical University, Xi’an Shaanxi, China.

**Keywords:** angiotensin receptor blockers, angiotensin-converting enzyme inhibitor, atrial fibrillation, overview, prevention and cure

## Abstract

Background: Atrial fibrillation (AF) is a type of arrhythmia that represents a severe health hazard. The current therapies for AF have achieved success in some conditions. However, because the mechanisms underlying the occurrence and development of this disease remain unclear, the current treatment for AF often does not achieve the desired outcomes. Angiotensin-converting enzyme inhibitors (ACEIs) and angiotensin receptor blockers (ARBs), which exert robust effects on specific cardiovascular diseases, are widely used in the clinic. Several studies are focusing on the effect of ACEIs/ARBs on the prevention and cure of AF. Some systematic reviews have obtained different and even opposite results. An overview is required to obtain a conclusion and provide strong evidence to guide clinical work.

Methods: We searched 5 databases, including MEDLINE, EMBASE, Cochrane Library, Web of Science, and CNKI (Chinese), and selected relevant reviews that passed the assessment we performed. Then, we synthesized the data for each result from the included reviews and obtained conclusions.

Results: ACEIs/ARBs prevented new-onset AF and AF after heart failure. ACEIs/ARBs performed well in the prevention of secondary AF, especially postoperative AF. However, for patients suffering from hypertension and myocardial infarction, ACEIs/ARBs were not the right choices for preventing AF.

Conclusions: We suggest that physicians select ACEIs/ARBs as an anti-AF therapy for patients with heart failure due to their additional benefits. Moreover, for patients who have suffered AF, ACEIs/ARBs may be a routine drug for secondary prevention.

## 1 Introduction

Atrial fibrillation (AF) is the most common type of arrhythmia. Notably, the prevalence of AF increases with age.^[1]^ AF, a chronic disease with a high morbidity rate, places a substantial burden on patients and has considerable consequences for society.^[2]^ The demand for clinical prevention and treatment of AF is evident.

The current treatment strategies for AF mainly include anticoagulants, control of the heart rate, and management of the ventricular rate.^[3]^ With the development of ablation therapy and drugs, excellent progress has been achieved in AF treatment. However, a large number of patients are still unable to achieve their intended targets.^[4]^ Previous studies have reported that approximately 50% of patients undergoing repeated AF ablation experience recurrence.^[5]^ Thus, methods to prevent and cure AF effectively remain a puzzle that has attracted increasing attention from physicians.

The mechanism of AF occurrence is still not precisely understood. Imazio^[6]^ reported that AF is accompanied by myocardial and electrophysiological remodeling. Moreover, the renin-angiotensin-aldosterone system (RAAS) participates in myocardial and electrophysiological remodeling in the atria during AF development.^[7]^ For instance, a RAAS activator accelerates atrial fibrosis and facilitates myocardial hypertrophy. Drugs designed to inhibit the RAAS include angiotensin-converting enzyme inhibitors (ACEIs) and angiotensin receptor blockers (ARBs), which are first-line drugs used to treat cardiac diseases. ACEIs/ARBs inhibit the RAAS to delay heart remodeling in patients with AF.^[8]^ Thus, ACEIs/ARBs may theoretically prevent and cure AF.

The results of systematic reviews of randomized controlled trials (RCTs) are recognized as the highest-level evidence that assesses the effectiveness of interventions.^[9]^ Systematic reviews provide reliable evidence for doctors in guiding clinical practice. Researchers also require this evidence to verify shared conclusions and establish decisions for further work. However, systematic reviews include RCTs of only one specific intervention for a particular disease or condition—an overview of reviews remedies this shortcoming of systematic reviews. Overviews summarize the conclusions of different interventions for the same disease or condition and the same intervention for different diseases or conditions. This overview summarizes the evidence from systematic reviews on the effectiveness of ACEIs/ARBs in preventing and curing AF.

## 2 Methods

### 2.1 Criteria for including reviews

We included systematic reviews of RCTs that estimated the effects of ACEIs/ARBs on the prevention and cure of AF. We selected systematic reviews assessing ACEI/ARB interventions in patients with AF. The results from every patient included in those reviews were extracted separately. Reviews of every subtype of AF and every type of ACEI/ARB were included and the conclusion must be clear and complete (Table 1). We included not only the prevention and cure of AF but also the prevention of complications in the present study. We included reviews published in English and Chinese.

**Table 1:** Application of the PICO search strategy.

Population	Aged 18 or more who had risk for different types of AF
Intervention	Application of ACEI/ARB
Out come	Got AF or AF reappeared
Setting	Any healthcare setting
Study design	
	Systematic reviews that had explicitly searched for randomized controlled trials (RCTs); to be classified as a systematic review if the following criteria were met:
	1. Clear inclusion criteria
	2. A systematic search strategy
	3. A screening procedure to identity relevant studies
	4. Systematic data extraction and analysis procedures for RCTs

ACEIs = angiotensin-converting enzyme inhibitors, AF = atrial fibrillation, ARBs = angiotensin receptor blockers, PICO = population, intervention, comparison, outcome.

### 2.2 Exclusion criteria

The literature on the non-major intervention with ACEIs/ARBs in the treatment group, retrospective studies, repeated publications, comments, conference abstracts, and studies in which the data were unable to be extracted or were incomplete was excluded.

### 2.3 Search strategy

We (YZX and ZD) screened 5 databases, including MEDLINE, EMBASE, Cochrane Library, Web of Science, and CNKI from inception to July 25, 2020. We applied Boolean operators in the search strategy. We modified the search terms and strategy to meet the different requirements of each database. Additionally, we screened all references of the included articles to ensure that we collected as many related studies as possible.

### 2.4 Identifying relevant reviews and assessment of the methodological quality

Two independent investigators (YZX and ZD) assessed the relevance and methodological quality of all included articles according to the Assessing Methodological Quality of Systematic Reviews (AMSTAR) criteria,^[10]^ as shown in the checklist at https://amstar.ca/Amstar_Checklist.php. Discrepancies were handled by consultation and guidance from YF. We classified the studies into 3 levels according to the AMSTAR score: “with enormous limitations,” “with acceptable limitations,” and “with few limitations.” Articles with few limitations had scores of at least 8, those with acceptable limitations had scores of 4 to 7, and those meeting fewer than 3 criteria were labeled as “with enormous limitations.” We excluded reviews with enormous limitations.

### 2.5 Data extraction and synthesis

Two investigators (YZX and ZD) independently extracted data from each included study. We extracted the results using the patient intervention comparison and outcome (PICOS) in Table 1 and the baseline characteristics of every review. The methodological quality of RCTs in each review included was also extracted. The data synthesis was completed together.

### 2.6 Assessment of reviews

We applied principles from the Grading of Recommendation, Development, and Evaluation (GRADE) to assess the evidence we extracted. GRADE is a system that grades the quality of evidence for outcomes in reviews and the strengths of recommendations.^[11]^ The quality of evidence represents how confident other researchers are in accepting the results and conclusion. High-quality evidence indicates little possibility for change in further work. Low-quality evidence is not convincing and may change considerably in the future. Designs of primary studies, the quality of primary studies, consistency and directness were the components of judgments. According to the judgments, we divided the results in each review into four levels: “high,” “medium,” “low,” and “no evidence.” After grading the quality of evidence for each outcome in each comparison in each systematic review, the overall level of quality of the combined evidence was considered, as shown in Table 2.

**Table 2:** Characters of every systemic review.

Study	Number of included studies and participants	AMSTAR score	QR^∗^
Anand et al^[12]^	9 RCTs (N = 72,469)	9	QR: high
Chaugai et al^[13]^	26 RCTs (N = 165,387)	8	QR: high
Huang et al^[14]^	21 RCTs (N = 97,111)	10	QR: high
Kalus et al^[15]^	4 RCTs (N = 15,616)	6	QR: moderate
Zhao et al^[28]^	8 RCTs (N = 2323)	4	QR: moderate
Khatib et al^[16]^	14 RCTs (N = 92,817)	10	QR: high
Madrid et al^[17]^	7 RCTs (N = 24,849)	5	QR: moderate
Disertori et al^[19]^	8 RCTs (N = 4375)	6	QR: moderate
Han et al^[20]^	21 RCTs (N = 13,184)	9	QR: high
Healey et al^[21]^	11 RCTs (N = 56,308)	8	QR: high
Li et al^[22]^	15 RCTs (N = 3972)	8	QR: high
Chaugai et al^[26]^	4 RCTs (N = 1050)	6	QR: moderate
Schneider et al^[23]^	23 RCTs (N = 87,048)	10	QR: high
Jibrini et al^[24]^	26 RCTs (N = 102,005)	9	QR: high
Pan et al^[25]^	11RCTs (N = 55,971)	8	QR: high
Bhuriya et al^[29]^	3 RCTs (N = 27,885)	4	QR: moderate
Zhao et al^[30]^	22 RCTs (N = 2902)	7	QR: moderate
Chen et al (Medicine)^[27]^	6 RCTs (N = 53,510)	8	QR: high

∗ QR: quality of review, the maximum score on AMSTAR is 11 and scores.

## 3 Results

**Figure 1:**
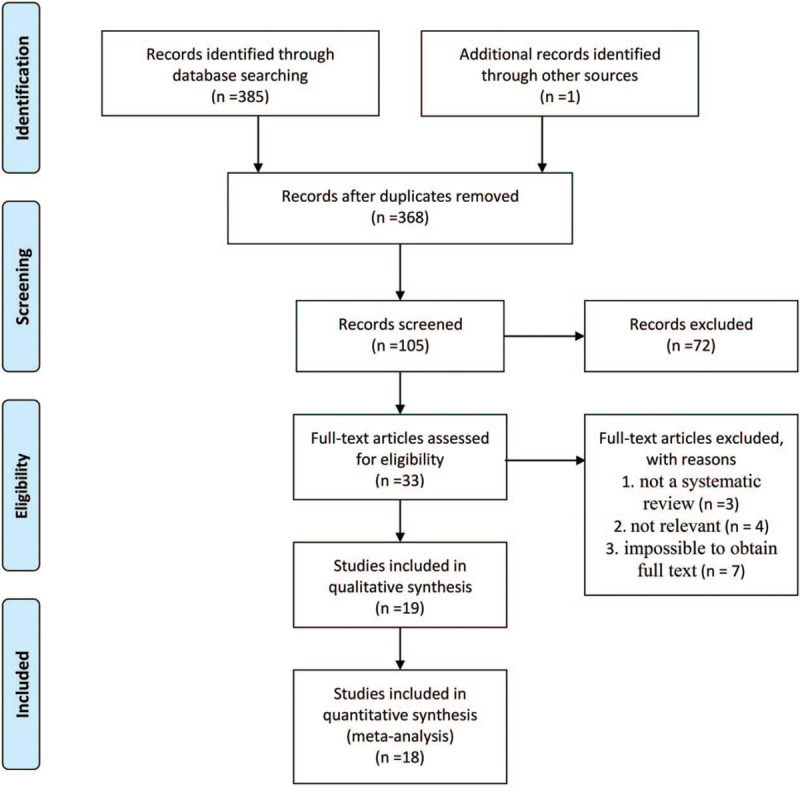
The workflow for selecting related meta-analyses.

The initial search identified 386 articles. After screening the titles and abstracts, the full texts of 33 systematic reviews were retrieved. We examined all passages and selected 18 reviews for this overview according to the criteria in Table 1. Reasons for the exclusion of 15 reviews were that the study was not a review (n = 3), not a relevant systematic review (n = 4), the full text was impossible to obtain (n = 7), and the study presented tremendous limitations in the methodological quality (n = 1) (Fig. 1). We listed the main characteristics and quality of the included reviews in Table 3.

**Table 3:** Quality of evidence.

Level of quality of evidence^∗^	Criteria
High-quality of evidence	One or more updated, high-quality systematic reviews that are based on at least 2 high-quality primary studies with consistent results
Medium-quality of evidence	One or more updated systematic reviews that are based on at least 2 primary studies of moderate quality with consistent results or 1 high-quality primary study
Low-quality of evidence	One or more systematic reviews of variable quality based on primary studies of moderate quality; inconsistent results in the reviews; inconsistent results in primary studies
No evidence	There is no systematic review identified on this topic

∗ Based on principles from Grading of Recommendations Assessment, Development, and Evaluation (GRADE).

This review primarily focused on the prevention of various AF types, including new-onset AF, recurrent AF, persistent AF, postoperative atrial fibrillation (POAF), and AF accompanied by different complications, such as AF with heart failure (HF), AF with hypertension, and AF after myocardial infarction (post-MI). Eleven reviews were of high quality, and the remaining 7 reviews were of medium quality.

### 3.1 Primary prevention

#### 3.1.1 New-onset AF

**Figure 2:**
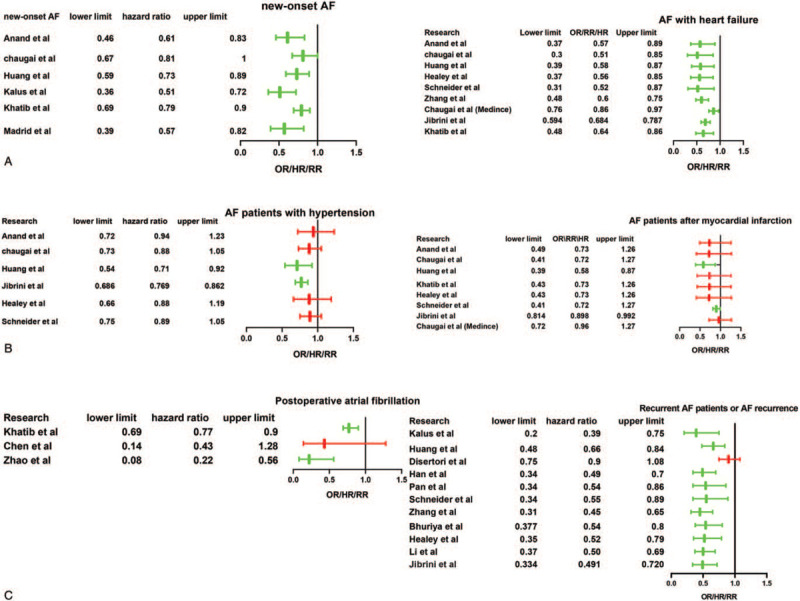
Forest plots for each included systemic review of (A) the new-onset AF subgroup, (B) AF with heart failure subgroup, (C) AF with hypertension subgroup, (D) AF post-MI subgroup, (E) POAF subgroup, and (F) recurrent AF or AF recurrence subgroup. The red forest plots present the results with *P*-values >.05. The green forest plot shows results with *P*-values <.05. AF = atrial fibrillation, MI = myocardial infarction, POAF = postoperative atrial fibrillation.

Six reviews reported the effects of ACEIs/ARBs on new-onset AF^[12–18]^ (Fig. 2A). Extensive RCTs assessed new-onset AF: 100 primary studies and 545,405 participants were included in the 6 reviews. In these systematic reviews, the intervention was ACEIs/ARBs, and the comparison was the placebo. These systematic reviews all concluded that ACEIs/ARBs reduced the incidence of new-onset AF. However, the RR was not very low, and the upper limit of the 95% confidence interval (CI) was close to 1. Therefore, ACEIs/ARBs reduced the incidence of new-onset AF, but the effect was not outstanding. For new-onset AF, ACEIs/ARBs might be an ancillary drug. In the overview, Zhang et al^[18]^ and Huang et al^[14]^ reported the same results in a population with new-onset AF (OR = 0.80; 95% CI, 0.70–0.92), although this result might be attributed to the substantial overlap in the RCTs selected.

#### 3.1.2 AF with heart failure

Nine reviews mentioned the effects of ACEIs/ARBs on AF with heart failure (HF) ^[12–14,18–23]^ (Fig. 2B). All reviews reported that ACEIs/ARBs slowed the overall development of AF. Chaugai et al^[13]^ performed further subgroup analyses and found that for patients with AF complicated with systolic heart failure, ACEIs/ARBs were associated with a 49% reduction in the AF incidence (OR: 0.51, 95% CI: 0.30–0.85, *P* = .01). However, a significant difference was not observed in the incidence of diastolic heart failure (OR: 1.02, 95% CI: 0.80–1.31, *P* = .877). Zhang et al^[18]^ divided the subgroups according to the left ventricular ejection fraction (LVEF). They found that among patients with AF and an LVEF ≥40%, ACEI/ARB users had a significantly lower risk of new-onset AF compared with nonusers (OR: 0.86; 95% CI: 0.76–0.96). For patients with LVEF < 40%, ACEI/ARB users had a significantly lower risk of AF than nonusers (OR: 0.60; 95% CI: 0.48–0.75). The included reviews all reported an ideal decrease in the risk of AF occurrence in patients with HF. The results illustrated that patients with HF could select ACEIs/ARBs as their first choice due to their additional benefits in the prevention of AF.

#### 3.1.3 Patients with AF complicated with hypertension

Many systematic reviews have focused on patients with AF complicated with hypertension (Fig. 2C). However, we were unable to easily draw a conclusion on the effect of ACEIs/ARBs on patients with AF complicated with hypertension. According to 4 reviews, a significant reduction in AF was not observed in patients with hypertension.^[12,13,21,23]^ Madrid et al^[17]^ found a more significant difference in the pooled development of AF after excluding hypertension trials. These 4 reviews were a dissenting voice against the selection of ACEIs/ARBs to cure and prevent AF in patients with hypertension. Chaugai et al^[13]^ performed a further subgroup analysis and found that RAAS blocker therapy was associated with a 34% reduction in the incidence of persistent AF in patients with hypertension presenting with persistent AF (OR: 0.66, 95% CI: 0.45–0.95, *P* = .027). The result was opposite their findings for overall AF. In the other 2 reviews,^[14,24]^ the authors documented a positive effect of ACEIs/ARBs on the prevention of AF in patients with hypertension. Pan et al^[25]^ compared the effect of telmisartan on AF recurrence to other antihypertensive drugs. Telmisartan produced an ideal result (HR: 0.54, 95% CI 0.34–0.86, *P* < .05). The RRs in those 2 reviews were not very low, suggesting that ACEIs/ARBs did not reduce enormous risks of the development of AF.

#### 3.1.4 Patients with AF after MI

Seven reviews including 115 RCTs discussed AF after MI^[12–14,16,21,23,24,26]^ (Fig. 2D). Most (6 of 8) reported the definite conclusion that ACEIs/ARBs did not cure or prevent post-MI AF. Moreover, Khatib et al^[16]^ evaluated the effect of ACEIs/ARBs on the prevention of AF in patients with high risk/CAD, and they reached the same conclusion that ACEIs/ARBs did not produce a significant reduction (RR = 0.90, 95% CI: 0.77–1.05). Only Jibrini et al^[24]^ and Huang et al^[14]^ considered ACEIs/ARBs to reduce the relative risk of AF after MI (RR = 0.898, 95% CI: 0.814–0.992 and OR = 0.58, 95% CI: 0.39–0.87, respectively)

#### 3.1.5 POAF

Every cardiologist is concerned about POAF. The occurrence of POAF represents an intractable complication of the operation and new clinical symptoms in patients with a complex case. Moreover, POAF confuses both patients and physicians. In this overview, POAF represents all AF surgeries, except electrical cardioversion. Three reviews included related research^[16,27,28]^ (Fig. 2E) and identified a significant reduction in the risk of POAF with the use of ACEIs or ARBs in patients who underwent aortic valve replacement and general thoracic surgery. According to Zhao et al,^[28]^ ACEIs reduced the risk of POAF by 78% compared with the placebo/usual care (OR = 0.22; 95% CI: 0.08–0.56). However, after coronary artery bypass graft, ACEIs/ARBs did not protect patients from POAF (OR = 1.04; 95% CI: 0.91–1.09).^[27]^

#### 3.1.6 Patients with recurrent AF or AF recurrence

Ten reviews reported the effects of ACEIs/ARBs on AF recurrence or recurrent AF^[14,15,18–25,29]^ (Fig. 2F). All the reviews, except for the review by Disertori et al,^[19]^ concluded that ACEIs or ARBs significantly reduced the incidence of recurrent AF or AF recurrence. Disertori et al^[19]^ performed a subgroup analysis for ACEIs and ARBs. They drew an interesting conclusion that ACEIs and ARBs exerted different on the prevention of secondary AF. ACEIs exerted positive effects and ARBs did not have a significant effect on the prevention of secondary AF. In this analysis, due to the larger number of participants in the ARB subgroup than the ACEI subgroup (323–3567), we selected the results from the ARB subgroup to assess their conclusions. However, substantial heterogeneity was observed in the ARB subgroup compared with the ACEI subgroup (*I*^2^ = 79%, *P* < .05 vs *I*^2^ = 0, *P* = .72), indicating that we needed to be cautious in accepting the conclusion. The RR results all reached an excellent level. Moreover, these 10 reviews were all based on at least medium-quality primary studies, including a large number of participants. We had sufficient evidence to determine that the conclusion would not change substantially in the future.

## 4 Discussion

We performed a thorough documentation retrieval, assessment of both primary studies and reviews, and synthesis of the results from the included reviews in this overview of systematic reviews. Few overviews have focused on the prevention of AF, and no overview similar to ours has discusses the effects of ACEIs/ARBs on AF at the time this manuscript was completed.

We included 18 reviews in the overview, most of which (17 of 18) focused on the cure and prevention of AF. We analyzed the effect of ACEIs/ARBs on 2 main groups (primary and secondary prevention) and 5 subgroups (new-onset AF, AF with heart failure, AF with hypertension, recurrent AF or AF, recurrence, and POAF).

After evaluating the reviews included in this overview, only a few comparisons could be treated as high-quality evidence, due to the complexity of AF and its complications. The conclusion we drew in this overview about patients with AF complicated with hypertension was different from the suggestions offered by specialists in the 2016 ESC Guidelines.^[8]^ The few high-quality studies we included in this study might have caused this discrepancy.

Overall, ACEIs/ARBs exert a positive effect on the cure and prevention of AF. The same conclusion was confirmed for some complications of AF. Chaugai et al^[26]^ extensively analyzed a series of events after AF and concluded that ACEIs/ARBs reduced the incidence of cardiovascular events (OR: 0.83, [95% CI: 0.70–0.99], *P* = .038), especially heart failure (OR: 0.86, [95% CI: 0.76–0.97], *P* = .018). However, we do not recommend that all patients with AF should select ACEIs/ARBs as their antiarrhythmic drugs. In primary prevention, patients with new-onset AF obtained benefits in our overview. Nevertheless, ACEIs/ARBs did not benefit them much in reducing the incidence compared with the placebo. These patients might benefit more from current antiarrhythmic drugs. Considering their additional effects on AF, ACEIs/ARBs might be an ancillary drug or the first choice for other cardiovascular events. In a further subgroup analysis, we found that AF with heart failure was the best indication for the application of ACEIs/ARBs. ACEIs/ARBs are one of the current drugs used for patients with heart failure. Our overview provided persuasive evidence that the selection of ACEIs/ARBs for patients with heart failure is a wise choice. However, for patients with AF complicated with hypertension or post-MI AF, ACEIs/ARBs did not show ideal effects on the prevention of AF. The reason for this difference requires further fundamental and clinical research. This overview suggests that physicians should use other current antiarrhythmic drugs to prevent AF in patients with hypertension or MI.

In the secondary prevention of AF, ACEIs/ARBs performed wonderfully. ACEIs/ARBs prevented recurrent AF and AF following cardioversion and reduced the risk to a superficial level, which is good news for every cardiologist. The cardiologist could even apply ACEIs/ARBs as a routine drug for a patient with AF who has received anti-AF treatment. Han et al^[20]^ reported that the application of ACEIs/ARBs combined with an AAD, such as amiodarone, would achieve a better outcome (OR = 0.37; 95% CI, 0.29–0.48; *P* < .00001). Only Disertori et al^[19]^ declared that ARBs were not effective at preventing AF recurrence, but the study had substantial heterogeneity. In the same study, the authors found that ACEIs performed well in preventing AF recurrence. This result reminded us that the combined usage of ACEIs/ARBs with other drugs might yield surprising results in some patients with refractory AF.

## 5 Limitations

This overview had 2 main limitations. First, primary studies were overlooked. We observed extensive overlap among primary studies in the reviews. The systematic reviews covering the same RCTs reached similar conclusions, producing deviation in the synthesis of results. The second limitation is that the classification was unclear. Reviews have limited information about the trials, and the conclusions may become too broad to be useful for clinicians. The last limitation is that the analytical methods were not quantified, which led to inescapable error in the overview.

## 6 Conclusions

We listed the effects of ACEIs/ARBs on each condition and the quality of all evidence in Table 4.

**Table 4:** Summary of findings for quality of evidence across systematic reviews.

Disease to cure or prevent	Comparison	Results	Quality of evidence
AF	Placebo or conventional therapy	ACEI/ARB could prevent the AF, overall	Medium
New-onset AF	Placebo or conventional therapy	ACEI/ARB could prevent the new-onset AF	Medium
AF with heart failure	Placebo or conventional therapy	ACEI/ARB could prevent the AF with heart failure	High
AF patients with hypertension	Placebo or conventional therapy	ACEI/ARB could not prevent the AF with hypertension	Low
AF patients after myocardial infarction	Placebo or conventional therapy	ACEI/ARB could not prevent the AF after myocardial infarction	High
Secondary prevention	Placebo or conventional therapy	ACEI/ARB did well in secondary prevention of AF, overall.	High
Recurrent AF patients	Placebo or conventional therapy	ACEI/ARB could prevent the recurrent AF	High
Postoperative atrial fibrillation	Placebo or conventional therapy	ACEI/ARB could prevent the POAF	high

ACEIs = angiotensin-converting enzyme inhibitors, AF = atrial fibrillation, ARBs = angiotensin receptor blockers, POAF = postoperative atrial fibrillation.

In the overview, we concluded that ACEIs/ARBs should be used to prevent AF in patients with heart failure. Moreover, patients with a history of AF should receive ACEI/ARB treatment due to its fantastic performance in the secondary prevention of AF. ACEIs/ARBs are representative drugs that require more research before their further application in the clinic.

## Acknowledgments

All authors declare that the work described is original research that has not been published previously, and not under consideration for publication elsewhere, in whole or in part. All the authors listed have approved the description of the results.

## Author contributions

**Project administration:** Fu Yi.

**Supervision:** Qiuhe Ji, Fu Yi.

**Visualization:** Zhixiang Yu.

**Writing – original draft:** Zhixiang Yu, Dong Zhang.

**Writing – review & editing:** Qiuhe Ji, Fu Yi.
